# *Numb* downregulation suppresses cell growth and is associated with a poor prognosis of human hepatocellular carcinoma

**DOI:** 10.3892/ijmm.2015.2279

**Published:** 2015-07-09

**Authors:** CHENGZHI XIE, ZHENHUI LU, GUOXING LIU, YU FANG, JIEFENG LIU, ZHAO HUANG, FUSHENG WANG, XIAOLONG WU, XIAOHUA LEI, XIAOCHENG LI, YUEMING ZHANG, ZECHENG HU, KE QIAN, JIXIONG HU, SHENGFU HUANG, DEWU ZHONG, XUNDI XU

**Affiliations:** 1 Division of Hepatobiliary-Pancreatic Surgery, Department of Surgery, The Second Xiangya Hospital, Central South University, Changsha, Hunan 410011, P.R. China; 2 Department of General Surgery, The Second Affiliated Hospital, Hunan University of Chinese Medicine, Changsha, Hunan 410005, P.R. China; 3 Department of Hepatobiliary Surgery, General Hospital of Ningxia Medical University, Yinchuan, Ningxia Hui Autonomous Region 750004, P.R. China

**Keywords:** hepatocellular carcinoma, *Numb*, proliferation, prognosis, apoptosis

## Abstract

Numb, an endocytic adaptor, is a known cell fate determinant that participates in asymmetric cell division. The present study aimed to explore the potential roles of *Numb* in hepatocarcinogenesis. *Numb* expression was investigated in hepatocellular carcinomas (HCC) with reverse transcription-quantitative polymerase chain reaction and immunohistochemical examination; its association with the prognosis of HCC patients was analyzed. In addition, the effects of *Numb* deletion on proliferation of HCC cells and its relevant molecules were evaluated in Huh7 and HepG2 cells. *Numb* overexpression was observed in 62% of adjacent non-tumor tissues and 46% of tumor tissues. Overexpression of *Numb* in HCC was associated with histological grade, portal vein invasion and the number of tumors (P=0.001, 0.022 and 0.034 respectively). Multivariate analysis revealed that *Numb* expression was an independent prognostic indicator of HCC patients. Methylation of the *Numb* promoter contributed to hepatocarcinogenesis. *In vitro* assays demonstrated that *Numb* silencing resulted in inhibition of cell proliferation, induction of apoptosis, down-regulation of cyclin-dependent protein kinase 4 (CDK4) and S-phase kinase-associated protein 2 (SKP2), and upregulation of Bcl-2 homologous antagonist/killer (BAK) and cyclin-dependent kinase inhibitor 1 (p21). The present study suggests that downregulation of *Numb* inhibits colony formation and cell proliferation, induces apoptosis of HCC cells and independently predicts the poor prognosis of HCC patients. Thus, *Numb* has a potential role in the development and progression of HCC.

## Introduction

Primary liver cancer, which consists predominantly of hepatocellular carcinoma (HCC), is the sixth most common cancer worldwide and the second most common cause of cancer mortality (1). HCC accounts for 70 to 90% of primary liver cancers. There is an increasing understanding of the molecular mechanisms that induce hepatocarcinogenesis, including abrogation of cell-cycle checkpoints, and activation of oncogenic pathways and Notch (2–4). Further detailed clarification of the molecular pathways involved in hepatocarcinogenesis could improve the treatment of HCC patients.

Numb, a membrane-associated protein, asymmetrically localizes during the division of neuroblasts and subsequently segregates to one daughter cell at telophase, where it functions as an intrinsic determinant of cell fate (5–7). *Drosophila* and vertebrate *Numb* genes have a role in binary cell fate decision in the peripheral and central nervous system (8–9). Intrinsically inherited *Numb* interacts with the cell surface receptor Notch and a serine-threonine kinase, *Numb*-associated kinase (Nak), and functions, at least in part, by antagonizing Notch activity (10–11). Subversion of *Numb* is associated with important human pathologies, including cancer. An *in vivo* RNA interference screen in a model of mouse lymphomagenesis identified *Numb* as a putative tumor suppressor, whose ablation can accelerate the onset of lymphomas (12). In breast cancer there is a frequent loss of *Numb* expression, which causes increased activity of the receptor Notch (13). Furthermore, *Numb* enters in a tricomplex with p53 and the E3 ubiquitin ligase, HDM2, thereby preventing ubiquitination and degradation of p53, and results in increased p53 protein levels and activity (14). The present study was designed to assess the role of *Numb* in liver carcinogenesis.

## Materials and methods

### Patients and follow-up

Fresh tumor samples and corresponding non-tumor tissues used in reverse transcription quantitative-polymerase chain reaction (RT-qPCR) and western blot analyses were randomly collected from HCC patients who underwent curative resection between 2006 and 2009 in The Second Xiangya Hospital (Central South University, Hunan, China). All the specimens were removed and preserved as described (15). Tumor specimens used in immunohistochemistry were obtained from 85 HCC patients (62 males and 23 females, 28–72 years of age) who underwent hepatectomy in the department. All these patients had hepatitis B virus infection. The median follow-up period of these patients was 34 months (range, 1–78 months). Previous informed consent was obtained, and the study protocol was approved by the Ethics Committee of The Second Xiangya Hospital.

### Cell lines

Huh7 and HepG2 cell lines were purchased from the certified biological resources center China Center for Type Culture Collection (Wuhan, China). These cell lines were maintained at 37°C in a humidified incubator under 5% CO_2_ conditions, as described previously (2,16).

### Immunohistochemistry

A total of 85 samples of HCC and their corresponding non-tumoral liver tissue were assessed by immunohistochemistry. Immunostaining was performed as described previously (17). Polyclonal rabbit anti-human Numb (sc-25668, 1:100 dilution; Santa Cruz Biotechnology, Inc., Dallas, TX, USA) was used to detect the expression of Numb.

### Methylation-specific PCR (MSP), RT-qPCR and western blot analyses

RT-qPCR of 13 HCC samples and related non-tumor counterparts were analyzed as described previously (17). The primers are exhibited in Table I. For other analyses, RNA was collected from cultured cells with the TRIzol reagent (Invitrogen Life Technologies, Carlsbad, CA, USA), according to the manufacturer's instructions.

Nine HCC samples and the related non-tumor-counterparts were further used in western blot analysis as described previously (15). The following primary antibodies were used in western blot analysis: Rabbit polyclonal antibodies against Numb, cyclin-dependent protein kinase 4 (CDK4) (sc-260), S-phase kinase-associated protein 2 (SKP2) (sc-7164), Bcl-2 homologous antagonist/killer (BAK) (sc-832), cyclin-dependent kinase inhibitor 1 (p21) (sc-397) and GAPDH (sc-25778) (all from Santa Cruz Biotechnology, Inc.).

Thirty primary HCC tumors and their paired non-tumorous tissues were investigated by MSP, as described previously (18).

### Cell proliferation, colony formation, cell cycle and apoptosis assay

The functional role of *Numb* in HCC cells was analyzed with small interfering RNA (siRNA). The *Numb* siRNA sequence was UGGAACAUAAACAUCCUUCUUUCUC. The negative control siRNA (cat. no. 12935-200; Invitrogen Life Technologies) was used as the negative control for all the experiments. The transfection of siRNA into HepG2 and Huh7 was performed with Lipofectamine 2000 (cat. no. 11668-019; Invitrogen Life Technologies) according to the manufacturer's instructions.

Cell proliferation, colony formation, cell cycle and apoptosis assays were performed as described previously (16).

### Statistical analysis

Statistical analyses were performed with SPSS 16.0 for Windows (SPSS, Inc., Chicago, IL, USA). Cumulative survival time was calculated by the Kaplan-Meier method and analyzed by the log-rank test. Univariate and multivariate analyses were based on the Cox proportional hazards regression model. The χ^2^ test, Fisher's exact test and Student's t-test were used for comparison between groups. All the tests were two-tailed. Values of P<0.05 were considered to indicate a statistically significant difference.

## Results

### Numb expression in HCC

**Figure 1:**
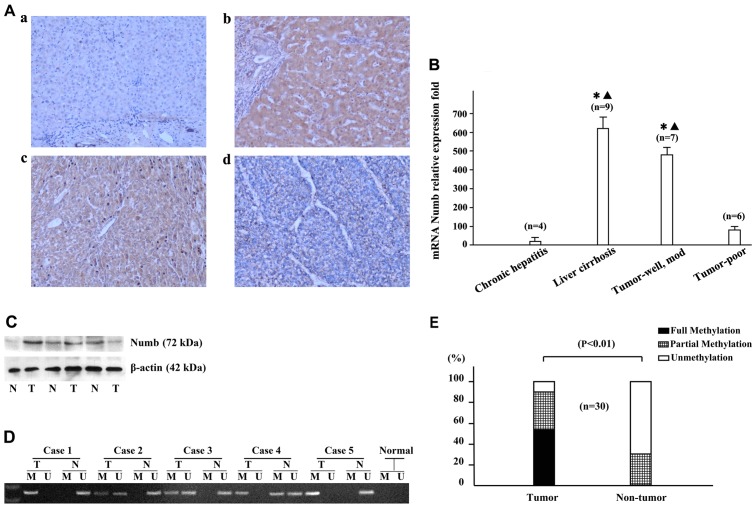
Expression of Numb in samples from hepatocellular carcinomas (HCC) patients. (A) Expression of Numb in non-tumorous and tumor liver tissues detected by immunohistochemistry; (a) chronic hepatitis, (b) liver cirrhosis, (c) moderately differentiated HCC, (d) poorly differentiated HCC. Magnification, ×100. (B) Reverse transcription-quantitative polymerase chain reaction (PCR) was used to examine *Numb* mRNA expression from 13 paired HCC tissues and their non-tumor counterparts. The results (mean ± standard deviation) were normalised for β-actin expression. Statistical analysis was performed comparing liver cirrhosis, well- and moderately differentiated tumors, and poorly differentiated tumors with chronic hepatitis; ^*^P<0.05; and liver cirrhosis, and well-and moderately differentiated tumors compared with poorly differentiated tumors; ^▲^P<0.05. (C) The representative expression of Numb as assessed by western blot analysis. Lane 1, chronic hepatitis B; lanes 2 and 4, moderately differentiated tumors; lanes 3 and 5, liver cirrhosis; lane 6, poorly differentiated tumor. (D) Methylation of *Numb* promoter in HCC tissues was determined by methylation-specific PCR (MSP). (E) MSP showed that the methylation of *Numb* was significantly detected in HCC tissues (T) compared with the corresponding non-tumorous tissues (N); n=19, P<0.01. M, methylated DNA; U, unmethylated DNA.

Cellular localization of the Numb protein was assessed by immunohistochemistry in the tumor tissue and their corresponding non-tumoral liver tissue from 85 samples of HCC. In general, high expression of the Numb protein was observed in both tumor tissue (39/85, 46%) and non-tumoral liver tissue (53/85, 62%), and the staining intensity varied widely. In normal liver and chronic hepatitis samples, Numb expression was low (Fig. 1A-a). The majority of cirrhotic liver samples showed increased *Numb* expression, particularly in regenerative nodules (Fig. 1A–b). High expression of the Numb protein was significantly more frequent in the samples of cirrhotic liver (49/58, 84%) compared with the normal liver (0/3, 0%), chronic hepatitis (4/24, 17%) and cancer tissues (39/85, 46%), as shown in Table II. In the cancer tissues, the histopathological type of HCC was associated with Numb expression; the expression level decreased with poor cell differentiation. The majority of well- and moderately differentiated HCCs showed high Numb expression (36/46, 78%) (Fig. 1A–c), while poorly differentiated HCCs showed a low Numb expression (36/39, 92%) (Table III; Fig. 1A–d). The high expression of Numb was more frequent in liver cirrhosis and well-differentiated HCCs compared with the normal liver, chronic hepatitis or poorly differentiated HCCs.

RT-qPCR analysis was performed by 13 paired non-tumor and tumor mRNA extracts. *Numb* mRNA expression levels in the tissues of liver cirrhosis (n=9), well- and moderately differentiated (n=7) and poorly differentiated liver tumors (n=6) had 41-, 38- and 2-fold increased expression with respect to chronic hepatitis (n=4) (P<0.05 respectively). Additionally, decreased expression levels were observed in poorly differentiated liver tumors compared with those in the tissues of liver cirrhosis, and well- and moderately differentiated liver tumors (P<0.05 respectively) (Fig. 1B). In addition, overexpression of *Numb* was further confirmed by a western blot analysis in 6 cases (Fig. 1C).

### Aberrant methylation of Numb in HCC

The methylation frequency of *Numb* was investigated in 30 primary HCC tumors and their paired non-tumorous tissues by MSP (Fig. 1D).

In the tumor tissues, fully, partially or unmethylated alleles of *Numb* were detected in 16/30 (53.3%), 11/30 (36.7%) and 3/30 (10%) cases, respectively, whereas in the paired non-tumorous tissues, 0/30 (0%), 9/30 (30%) and 21/30 (70%) cases were identified correspondingly, which supported that methylation of *Numb* has a role in hepatocarcinogenesis (Fig. 1E).

### Numb expression and its association with clinicopathological features and prognosis of the HCC patients

As shown in Table III, *Numb* expression in tumor samples was significantly associated with histological grade (P=0.001), portal vein invasion (P=0.022) and number of tumors (P=0.034) in the HCC patients. However, no statistically significant association was identified between *Numb* expression and the other clinical characteristics.

To further evaluate the prognostic value of *Numb* for HCC patients, univariate and multivariate analyses were performed for the clinicopathological characteristics and expression of *Numb*. In the univariate analysis, tumor differentiation, portal vein involvement and number of tumors were revealed to be associated with disease-free and overall survival of the HCC patients. No significant prognostic significance was identified in the other characteristics, including gender, age and capsular formation of patients for overall or disease-free survival (Table IV).

**Figure 2:**
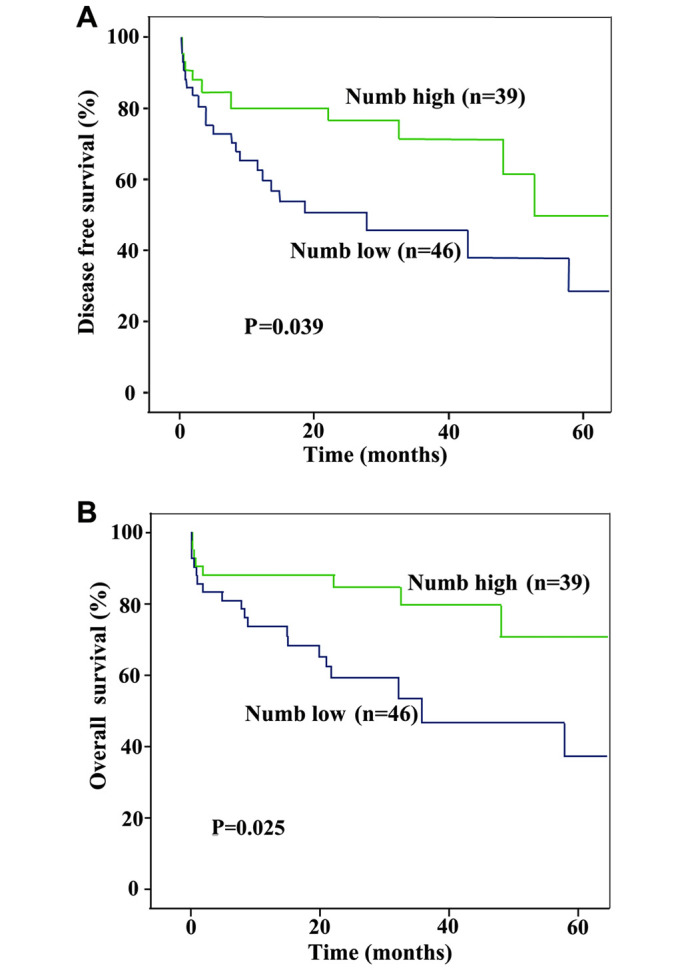
Kaplan-Meier analysis of disease-free and overall survival in 85 cases based on *Numb* expression. Compared with the group with low *Numb* expression, (A) the disease-free and (B) overall survival were significantly higher in the group with high *Numb* expression.

Individual features that showed significance by univariate analysis were adopted as covariates in a multivariate Cox proportional hazards model and subsequently the combined variables were further analyzed. *Numb* was shown to be an independent prognostic indicator for disease-free (P=0.039) and overall survival (P=0.025) (Fig. 2A and B).

### Antiproliferation of HCC cells by Numb silencing

Tumor suppression was assessed by soft agar formation and cell growth assay.

**Figure 3:**
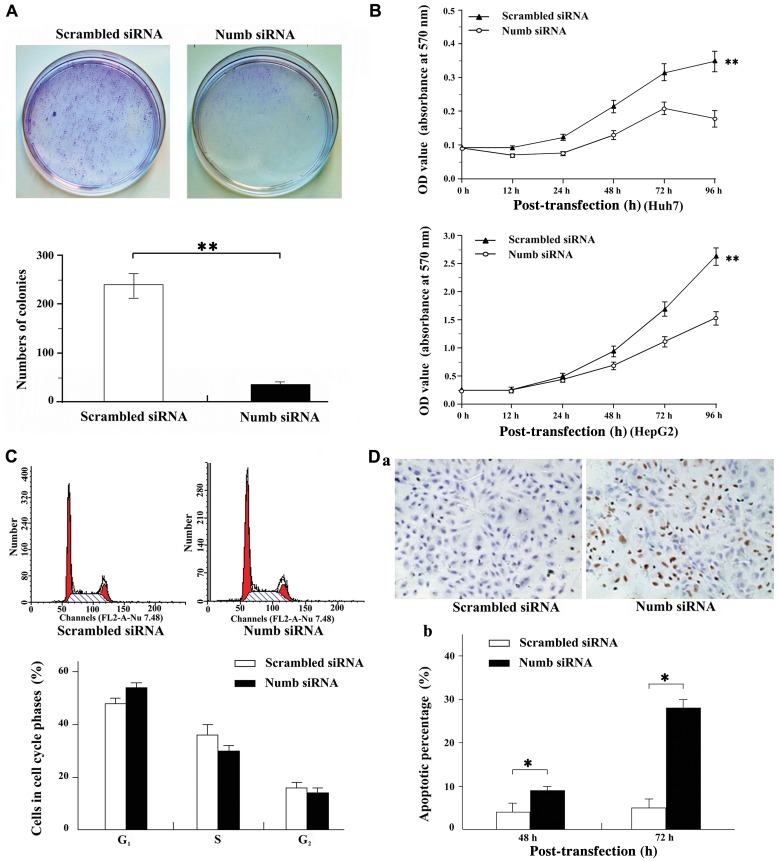
Oncogenetic potential of Numb in hepatocellular carcinomas (HCC). (A) As shown by representative dishes, Numb depletion significantly inhibited the colony formation of Huh7 cells in soft agar culture. Quantitative analyses of colony numbers are shown in the lower panel. Values are the mean ± standard deviation (SD) of ≥3 independent experiments. (B) MTT assay was performed in Huh7 and HepG2 cells. Growth curves of *Numb* small interfering RNA (siRNA) cells were compared with the scramble cells, respectively. The results are expressed as mean ± SD of ≥3 independent experiments. (C) Flow cytometric analysis showing that *Numb* siRNA for 24 h increased the G_0_-G_1_ phase fraction and decreased the S phase fraction when compared to the scramble treated cultures in Huh7 cells. (D-a) Representative images of TUNEL staining. A larger quantity of apoptotic cells were detected following treatment of Huh7 cells with *Numb* siRNA in comparison with that of the scramble cells. (D-b) The apoptotic index was compared between *Numb* siRNA and scramble cells (right panel).^**^P<0.01, ^*^P<0.001.

The soft agar assay showed that the frequency of colony formation of Huh7 cells was significantly inhibited (238.67±24.97 vs. 35.00±3.64, P<0.01) in *Numb* silencing compared with scramble cells (Fig. 3A).

*Numb* silencing also significantly inhibited the cell growth of Huh7 and HepG2 cells by the MTT assay. The statistical significance (P<0.01) between cultures treated with *Numb* siRNA versus scramble is shown in Fig. 3B.

### Induction of apoptosis by ectopic expression of Numb

To explore the molecular mechanism of *Numb* in HCC development, the role of *Numb* in the cell cycle was investigated with flow cytometry. In the Huh7 cells tested, treatment with *Numb* siRNA for 24 h increased the G_0_–G_1_ phase fraction and decreased the S phase fraction when compared with the scramble-treated cultures. A representative result is shown in Fig. 3C with the following fraction values for cultures treated with *Numb* siRNA versus scramble (G_0_–G_1_: 54.35 vs. 48.91; S phase fraction: 31.22 vs. 35.87; G_2_-M: 14.44 vs. 15.22, respectively).

The apoptotic index was compared between *Numb* siRNA and scramble cells by TUNEL staining. The TUNEL assay revealed that apoptotic cells treated by *Numb* siRNA were significantly higher compared with those of the scramble control (48 h: 4.39 vs. 21.94%; 72 h: 4.47 vs. 36.10%, respectively, P<0.001; Fig. 3D).

**Figure 4:**
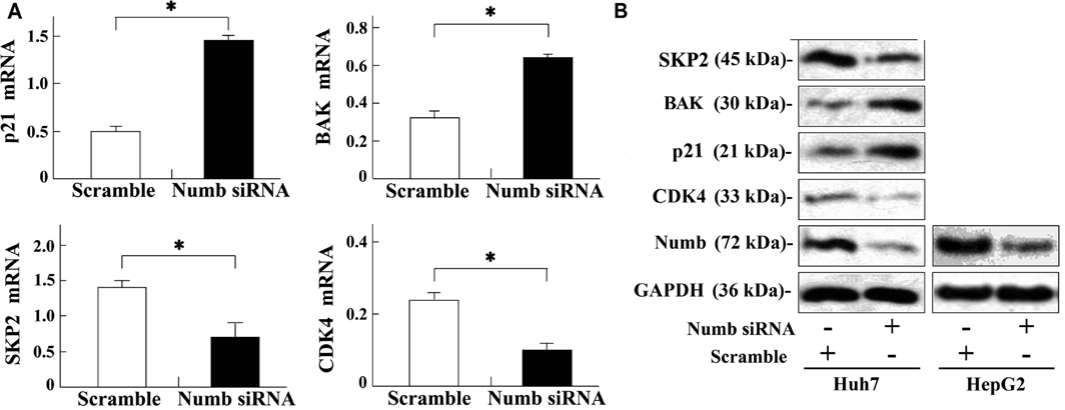
*Numb* inhibition inducing upregulation of cyclin-dependent kinase inhibitor 1 (*p21*) and Bcl-2 homologous antagonist/killer (*BAK*), and downregulation of cyclin-dependent protein kinase 4 (*CDK4*) and S-phase kinase-associated protein 2 (*SKP2*). (A) *Numb* small interfering RNA (siRNA) increased *p21* and *BAK* expression, and decreased *CDK4* and *SKP2* expression at the mRNA level by reverse transcription-quantitative polymerase chain reaction assays. Experiments were performed 24 (*p21*) and 48 h (*BAK*, *CDK4* and *SKP2*) after *Numb* siRNA transfection with Huh7 cells. (B) siRNA against *Numb* induced p21 and BAK expression, and suppressed CDK4 and SKP2 expression at the protein level. GAPDH served as a loading control. ^*^P<0.05.

To further elucidate the molecular basis of cell cycle and apoptosis induced by *Numb* silencing, the relevant molecules were screened by RT-qPCR and confirmed by western blot analysis. RT-qPCR analysis revealed that *CDK4* and *SKP2* were downregulated whereas *BAK* and *p21* were upregulated in the Huh7 cell line following *Numb* siRNA treatment (P<0.05, respectively) (Fig. 4A). Western blot analysis further confirmed these results (Fig. 4B).

## Discussion

In the present study, *Numb* was overexpressed in patients with HCC. A decreased expression of *Numb* in human HCC was an independent predictor of poor prognosis. *Numb* ablation by siRNA *in vitro* could inhibit tumor cell proliferation by downregulating *CDK4* and *SKP2* and upregulating *p21* expression, and enhancing the apoptotic potential by upregulating *BAK*.

The present results showed that the *Numb* gene was involved in hepatocarcinogenesis and its decreased expression was associated with a poor prognosis of HCC patients. In the non-tumor liver tissue, *Numb* was overexpressed in the majority of cirrhotic nodules and liver tissue infected with hepatitis B virus compared to the normal liver tissue. In addition, *Numb* was overexpressed in well- and moderately differentiated tumor tissues, which was similar to our previous reports on Vanilloid receptor-1, CB1 and CB2 receptors (17,19). Additionally, downregulation of *Numb* was associated with aggressive cancer phenotypes, including portal vein invasion and dedifferentiated histology (Table III). Univariate and multivariate analyses showed that a low expression of *Numb* was significantly associated with disease relapse and poor survival of HCC. These data indicate that *Numb* may serve as an independent predictor for the prognosis of HCC patients.

*Numb* is an evolutionary conserved protein that has been indicated in tumorigenesis with roles in controlling stem/progenitor cell development (20). In addition, a number of cellular processes, such as cell adhesion and migration controlled by *Numb*, are also involved in mammalian tumorigenesis, such as in chronic myelogenous leukaemia, breast cancer, non-small cell lung cancer and salivary gland carcinomas (13,14,21–24). In chronic myelogenous leukaemia, which progress from a slow-growing chronic phase to an aggressive blast crisis phase, high levels of *Numb* expression were observed in the chronic phase whereas low levels of *Numb* expression were observed in the blast crisis phase. In breast cancer, reduced expression of *Numb* correlating with decreased p53 levels and increased chemo-resistance was found to result in an aggressive tumor phenotype as illustrated by poor prognostic outcome for these patients (14,25). Concordant with these findings, a defect of *Numb* expression in HCC was associated with aggressive phenotypes of the tumor and poor outcome of the patients highlighted its involvement in hepatocarcinogenesis.

It has been well documented that DNA methylation of CpG islands located near gene promoters affects the transcription of specific genes (26,27). Epigenetic inactivation of genes by promotor methylation has been recognized as an important and alternative mechanism in carcinogenesis. In HCC, aberrant promoter methylation of p16 (*INK4*) is considered a main cause resulting in its function loss (28,29). In the present study, methylated alleles of *Numb* could be detected in 12/19 (63.2%) of tumor tissues, indicating the involvement of methylation in hepatocarcinogenesis.

On the basis of those findings above, the role of *Numb* was further explored in the proliferation of HCC cells. Compared with scramble RNA-treated HCC cells, *Numb* siRNA silencing resulted in the inhibition of proliferation and colony formation in soft agar, cell-cycle arrest and induction of apoptosis. These results suggest that *Numb* has an important role in the proliferative process of HCC cells.

Our previous molecular studies identified that *Numb* suppression could cause dysregulation of CDK4, SKP2, p21 and BAK, suggesting its roles in several signaling pathways. *Numb* is involved in p53, Notch and Hedgehog pathways in several tumors (14,30–32). The potential roles of cyclin-cdk complexes, such as CDK and cyclin kinase inhibitor families modulating in the G_0_–G_1_ phase of the cell cycle, have been addressed in human hepatocarcinogenesis (33,34). Progress has further shown that SKP2, which maintains and preserves the distinct phases during a cell cycle through protein degradation, has emerged in human hepatocarcinogenesis (35,36). The present data showed that *Numb* silencing resulted in upregulation of *p21* and downregulation of *CDK4* and *SKP2* in HCC cells, suggesting involvement of *Numb* in the G_1_-S phase of a cell cycle. In addition, BAK is a well-known cell death initiator in the apoptotic signaling cascade, and its roles in hepatocarcinogensis or for HCC treatment have also been documented (37,38). Different agents induce apoptosis in HCC cells by stimulating *BAK* expression, suggesting its pro-apoptotic effect (39,40). The present findings that upregulation of *BAK* was caused by *Numb* silencing highlights the role of *Numb* in apoptosis induction. Interactions among these molecules as targeted by *Numb* warrant further detailed investigation.

Based on the findings of the present study, it can be concluded that downregulation of *Numb* may be a predictor of HCC prognosis, and *Numb* has an important role in the proliferation of HCC cells *in vitro* via interaction with CDK4, p21, SKP2 and BAK. *Numb* may be a potential therapeutic target for HCC patients.

## Figures and Tables

**Table I:** DNA sequences of the primers used in the study.

Primer name	Sequence 5′→3′
RT-qPCR	
β-actin-F	ATCATGTTTGAGACCTTCAACA
β-actin-R	CATCTCTTGCTCGAAGTCCA
*Numb*-F	GGCATACAGAGGTTCCTACA
*Numb*-R	TGCTCCTTTGACCGCTAC
*BAK*-F	CAGGGCTTAGGACTTGGTTT
*BAK*-R	TTTTTTCAGGGTGAGGGGAT
*SKP2*-F	CTTTCTGGGTGTTCTGGATT
*SKP2*-R	GGAGCAATTAATCTGTAGATGAGG
*p21*-F	GCAGCGGAACAAGGAGT
*p21*-R	GGAGAAACGGGAACCAG
*CDK4*-F	CCCGAAGTTCTTCTGCAGTC
*CDK4*-R	GTCGGCTTCAGATTTCCAC
MSP	
*Numb* MSPm-F	TTTCGAAAGTGTTGGGATTATATAC
*Numb* MSPm-R	AACTACAATAAACCAAAATCGCG
*Numb* MSPu-F	TTGAAAGTGTTGGGATTATATATGT
*Numb* MSPu-R	AACTACAATAAACCAAAATCACACC

RT-qPCR, reverse transcription-quantitative polymerase chain reaction; F, forward; R, reverse; MSP, methylation-specific PCR; *CDK4*, cyclin-dependent protein kinase 4; *SKP2*, S-phase kinase-associated protein 2; *Bak*, Bcl-2 homologous antagonist/killer; *p21*, cyclin-dependent kinase inhibitor 1.

**Table II:** Immunohistochemical analysis of Numb in cancer and non-cancer liver tissues.

Pathological category	No.	Staining intensity for Numb, no.	
		Low	High
Normal liver	3	3	0
Chronic hepatitis	24	20	4
Cirrhosis	58	9^a^,^b^	49
HCC	85	46^c^	39

Intensity of Numb staining varied significantly according to liver disease state.

a Comparison with normal liver (P=0.007);

b with chronic hepatitis (P<0.001); and

c with cirrhosis (P<0.001). HCC, hepatocellular carcinomas.

**Table III:** Association between Numb expression and clinicopathological parameters in HCC.

Parameter	n	Expression of Numb, n		P-value
		Low	High	
Age, years				
≥60	55	24	21	
<60	30	22	8	NS
Gender				
Male	62	36	26	
Female	23	10	13	NS
Tumor size, cm				
<5	12	4	8	
*≥*5	73	42	31	NS
Histological grade				
Well/moderately differentiated	46	10	36	
Poorly/undifferentiated	39	36	3	0.001
Portal vein invasion				
Yes	24	16	8	
No	61	30	31	0.022
No. of tumors				
Solitary	58	31	27	
Multiple	27	15	12	0.034
Capsular formation				
Yes	36	21	15	
No	49	25	24	NS

Patients with hepatocellular carcinomas (HCC) were divided into Numb 'high' group (final density was higher compared with the median) and 'low' group (final density was lower compared with the median). The patient and disease profiles in each group were compared. NS, not significant between any groups.

**Table IV:** Univariate and multivariate analyses of factors associated with survival rate and recurrence.

Factors	Overall survival			Cumulative recurrence		
	Univariate	Multivariate		Univariate	Multivariate	
	P-value	HR (95% CI)	P-value	P-value	HR (95% CI)	P-value
Age, years						
<65	0.646	0.694	0.376	0.971	0.866	0.733
≥65		(0.349–2.753)			(0.378–1.981)	
Gender						
Male	0.504	0.980	0.970	0.607	0.979	0.968
Female		(0.340–2.647)			(0.346–2.772)	
*Numb* expression						
Low	<0.001	9.303	<0.001	<0.001	13.600	<0.001
High		(2.812–30.779)			(3.695–50.056)	
Tumor size, cm						
<3	0.681	0.925	0.598	0.856	0.950	0.906
≥3		(0.396–2.157)			(0.405–2.226)	
Tumor differentiation						
I/II	<0.001	0.349	0.013	<0.001	0.332	0.009
III/IV		(0.151–0.803)			(0.144–0.762)	
Tumor number						
Solitary	0.001	0.565	0.207	0.010	0.848	0.719
Multiple		(0.232–1.373)			(0.345–2.084)	
Portal vein invasion						
No	<0.001	0.403	0.041	<0.001	0.271	0.005
Yes		(0.168–0.964)			(0.110–0.671)	
Tumor encapsulation						
No	0.369	1.282	0.588	0.747	0.910	0.844
Yes		(0.521–3.156)			(0.359–2.311)	

HR, hazard ratio; CI, confidence interval.

## Abbreviations

HCC: hepatocellular carcinoma
BAK: Bcl-2 homologous antagonist/killer
CDK4: cyclin-dependent protein kinase 4
SKP2: S-phase kinase-associated protein 2
p21: cyclin-dependent kinase inhibitor 1
MSP: methylation-specific polymerase chain reaction
